# Tunnel construction in shallow soft rock using the pipe shed support

**DOI:** 10.1038/s41598-024-53634-8

**Published:** 2024-02-10

**Authors:** Liangliang Bao, Feng Wei

**Affiliations:** Institute of Architecture and Civil Engineering, Yulin University, No. 51 Chongwen Road, Yulin, 719000 Shaanxi China

**Keywords:** Civil engineering, Environmental impact

## Abstract

In order to clarify the impact mechanism of shallow buried soft rock tunnel excavation on the upper existing highway, as well as the mechanism of pipe shed reinforcement, a combination of theoretical analysis and on-site monitoring was used to conduct in-depth research on the Diantou Tunnel Crossing the existing highway project of Dayong Expressway. The impact of shallow buried soft rock tunnel crossing construction on the existing highway and safety control issues were studied, a new deformation control index, namely deformation difference rate, was introduced. The results show that the main lateral area of influence of rock deformation is within a distance of one diameter, and the overall area of influence is within a distance of twice the diameter. From the perspective of axial tunnel deformation, the deformation of surrounding rock tends to be stable when the excavation face passes through the monitoring section about twice the tunnel diameter. Effectively controlling rock deformation depends on the total amount of settlement deformation, the change rate of deformation, and the difference rate of deformation. For V-class shallow buried tunnel surrounding rock, in order to prevent cracks on the surface, the cumulative deformation of the surrounding rock needs to be less than 50 mm, the change rate needs to be less than 3 mm/day, and the difference rate needs to be less than 5 mm/m. Finally, the proposed control standard for surface subsidence is given for similar engineering reference.

## Introduction

The excavation conditions of shallow buried soft rock tunnels are extremely unfavorable, and problems such as poor self-stability of surrounding rock and excessive deformation of surrounding rock often occur after excavation. In addition, due to the influence of topographical and geological conditions and existing buildings and structures, a large number of new tunnels have been built in close proximity to the existing roads^1,2^, railways^3,4^, buildings^5,6^ and other special environmental, geological projects. During the construction of new tunnels, existing structures are often damaged. When the tunnel is shallower, and the surrounding rock is weakly broken, its construction is more complicated and riskier, which is highly prone to construction safety problems^7,8^. The deformation control standards of existing tunnels are generally determined by reference to relevant engineering experience and query specifications. Although the method has certain applicability, it is unreasonable. In engineering practice, the field test value often exceeds the control standard by a large margin but is still safe, resulting in a situation where no standard specification can be relied upon. In addition, the key construction step of tunnel construction also lacks corresponding control standard value^9,10^. Therefore, it is necessary to carry out further research on the deformation control method and control standard of shallow buried soft rock tunnels under the existing highway to meet the demand of tunnel engineering construction.

Through numerical simulation, indoor model tests, field monitoring, and theoretical analysis^11–20^, the influence of tunnel underneath construction on the settlement and deformation of existing pavement has been studied extensively. Combining the finite element numerical calculation method and on-site measured data, Li et al.^11^ established a modified Peck formula that can be used for calculating surface settlement caused by double-track tunnel excavation in combination with theoretical analysis methods. Liang et al.^12^ studied the influence of equivalent layer thickness and elastic modulus on surface subsidence by combining equivalent layer method and three-dimensional finite simulation. The results show that the thickness of the equivalent layer has a significant impact on surface subsidence, and surface subsidence is not sensitive to changes in elastic modulus. Sharifzadeh et al.^13^ used the three-dimensional finite element method to analyze the impact of tunnel excavation on surface settlement under different excavation sequences, and determined the optimal excavation sequence with minimum surface settlement. Li et al.^14^ analyzed the impact of shield construction in upper soft and lower hard composite strata on the surrounding environment using numerical calculation methods in combination with on-site monitoring data. Research shows that the maximum ground settlement occurs at a position where the soft rock height ratio (i.e. the ratio of the soft-rock height to the total height of the shield tunneling face) is 1.0. When the soft rock height ratio of adjacent excavation faces is between 0–0.2 and 0.5–1, shield construction can cause differential settlement. Lakirouhani et al.^15^ used a three-dimensional finite difference analysis method to study the impact of tunnel excavation and the presence of superstructure on land subsidence. The research shows that the number of superstructure layers has a greater impact on the maximum ground settlement compared to the free field situation. Cao et al.^16^ adopted the optimized double wall pilot tunnel method for tunnel excavation based on field tests and considering surface subsidence. The on-site monitoring results show that the optimized method is feasible. Pinto et al.^17^ derived a closed analytical solution of ground settlement caused by tunnel excavation using theoretical analysis methods based on the assumption of linear elasticity of soil mass. Suwansawat et al.^18^ considered the impact of construction factors, adopted Gaussian function to construct the settlement curve caused by double shield excavation construction, and combined the two curves to obtain the total ground settlement caused by double tunnel excavation. Ter-Martirosyan et al.^19^ used statistical analysis methods to study the impact of engineering geological conditions, surrounding architectural features, and tunnel construction parameters on surface subsidence. Research shows that engineering geological conditions and tunnel construction parameters have a significant impact on surface settlement, while the surrounding architectural features have little impact on surface settlement. Xu et al.^20^ established a prediction model for ground subsidence caused by excavating new tunnels under existing tunnels in heterogeneous strata based on the equivalent layered method and random medium theory, and verified the accuracy and applicability of the prediction model using numerical calculation methods. Based on the strength parameters derived from back analysis, Sharifzadeh et al.^21^ used the finite difference method to compare three different excavation sequences and optimize the excavation sequence. Daraei and Zare^22^ proposed a method to determine the sequence of tunnel excavation based on parameters such as failure strain, modified secant modulus, strength factor, and tunnel span. Song et al.^23^ optimized the blasting construction plan and proposed a two-step excavation method that is safe and fast. Song et al.^24^ proposed a new tunnel construction method—the double guide tunnel advanced construction method, and used numerical simulation method and on-site measurement method to verify the effect of this method in controlling the stability of surrounding rock. The results showed that this method is particularly suitable for tunnel construction in water-rich and weakly cemented sand layers.

The research mainly focuses on the settlement law and control standard of highway subgrade, the influence of different construction methods, and pre-reinforcement measures of shallow-buried tunnels under the highway on surface settlement, arch crown settlement, stress distribution, etc. On the basis of analyzing relevant specifications and standards, Tan et al.^25^ conducted some meaningful discussions on tunnel construction monitoring standards. Wang et al.^26^ provided safety control values and warning standards for underground comprehensive pipe gallery structures. Shi et al.^27^ established deformation control standards for the excavation construction on the side of the existing tunnel. Lai et al.^28^ suggested graded management of settlement control standards for existing subway lines, used 100% of the settlement control standard, 80% of the settlement control standard, and 60% of the settlement control standard as the control value (20 mm), alarm value (16 mm), and warning value (12 mm), respectively. Chen and Bai^29^ believed that the current ground settlement standards for tunnel crossings are unreasonable, as the characteristics of existing structures, geological conditions, and construction characteristics may not have been taken into account in the ground settlement standards. Though the previous studies have obtained favorable results to some extent, there is a highly complex relationship between tunnel excavation and the surrounding environment for the shallow buried soft rock tunnel underneath the existing project. The question arises of choosing an effective and feasible construction and support scheme considering the surrounding rock's geologic conditions and engineering environment. A constructive scheme to reduce the influence of the subterranean construction of the shallow buried soft rock tunnel on the existing engineering is required so that the tunnel can safely cross the complex geological section. The pipe-shed method is one of the important auxiliary methods for tunnel excavation, which has been widely used in the underground construction of shallowly buried soft rock tunnels and new tunnels through existing structures.

Scholars have explored the mechanical mechanism of pre-supporting the pipe-shed^30–39^. A double-layer pre-support system consisting of a pipe-shed and horizontal rotating-jet grout pile has been adopted in the Dongping No.1 Tunnel of the Guangfo Ring Railway in China^30^. Wu et al.^31^ proposed a pre-support technology combining pipe-shed and grouting reinforcement for loess tunnel excavation. Taking the tunnel crossing project of Taiyuan Railway Station as the background, Yang et al.^32^ studied the effect of advanced reinforcement of pipe-shed through large-scale similar model tests and engineering measurement data. Zhang et al.^33–35^ used the construction methods of freezing method, pipe-shed pre-support, and five-step excavation and fourteen-step excavation to excavate and construct super large cross-section tunnels in muddy soil layers, and used on-site test methods and numerical simulation methods to verify the effectiveness of the construction method. Xiao et al.^36^ proposed measures to improve the stability of the pipe-shed in combination with engineering practice and theoretical analysis methods. Hisatake et al.^37^ adopted centrifugal model tests to clarify the impact of pipe-shed pre-support and excavation methods on surface settlement. The research results show that the maximum ground settlement corresponding to the full-face excavation method with pipe-shed pre-support is one fourth of the maximum ground settlement corresponding to the full-face excavation method without pipe-shed pre-support. Nasir et al.^38^ used the finite difference method to compare the ground deformation caused by rectangular tunnels under pipe-shed pre-support and shield support. The results show that pipe-shed pre-support is suitable for soft soil layers, and shield support is suitable for soil layers with greater stiffness. Song et al.^39^ established a pipe-shed analysis model considering the integrity of the grouting zone based on the Winkler elastic foundation model, and verified the model using engineering measured data. The research results show that the effect of increasing the diameter of steel pipes on reducing deformation and internal force can be ignored, while reasonable steel pipe spacing and grouting amount can effectively reduce the deformation and internal force of the pipe-shed. However, due to the complexity of geotechnical engineering, many basic theoretical problems, for example, the mechanical mechanism of pipe shed in different types of soil, the influence mechanism of near structure construction under the reinforcement effect of pipe shed, and so on, have not been systematically studied and addressed. The research progress of the pipe-shed pre-support mechanism is difficult to meet the actual design and construction needs.

To clarify the mechanism of influence of excavation of shallow buried soft rock tunnels on existing highways, a comprehensive research method combining field monitoring and theoretical analysis is used. Relying on the Diantou Tunnel of Dayong Expressway under the existing highway project, the influence of the construction of existing highways under shallow buried soft rock tunnel and safety control of highways are studied. It is of great practical significance to ensure the construction safety of shallowly buried soft rock tunnels and the safety of existing highway projects, which can provide a new reference for the construction of similar tunnels in the future.

## Project overview

### Project background

**Figure 1:**
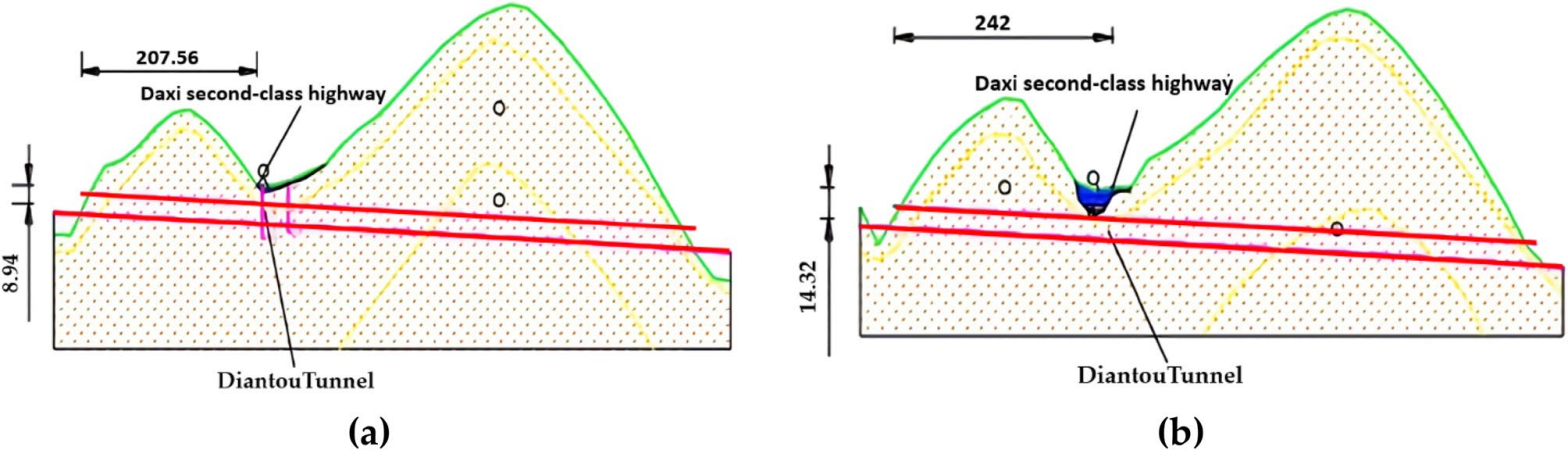
Schematic diagram of Diantou Tunnel undercrossing highway: (**a**) Left line; (**b**) right line.

**Figure 2:**
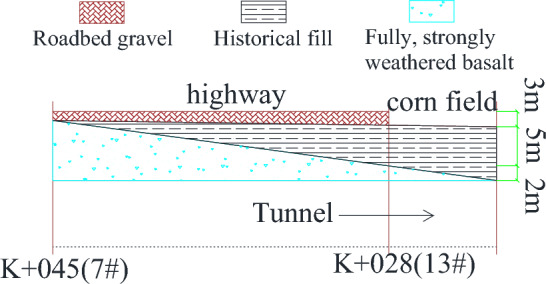
Tunnel axial—geological profile.

The Diantou Tunnel is an oversized section tunnel because its two-line tunnel sections have an area of up to 170 m^2^. As can be seen from Fig. 1a, b, both the left and right lines of the tunnel cross the Daxi Second-Class Highway at a very close distance, with the right tunnel crossing 14.32 m from the pavement, while the left tunnel crossing is only 8.94 m from the pavement. In addition, the actual gradient of the entrance section of the tunnel remains between 20°and 25°, while the actual gradient of the exit section remains between 30°and 35°, which shows that the formation and topography of the tunnel crossing zone have more apparent undulations. As shown in Table 1 and Fig. 2, the overlying rock (soil) layer on the tunnel crossing section is weak because it mainly comprises artificial fill, clay, full-weathered basalt, and strong-weathered basalt. The Diantou Tunnel is a two-way six-lane tunnel with a large section, which may cause significant deformation or collapse of the tunnel during actual construction.

**Table 1:** Main physical and mechanical indicators of rock and soil mass.

Name of rock and soil	State	Natural density (g/cm^3^)	Cohesion (kPa)	Internal friction angle (°)	Elastic modulus (GPa)	Poisson's ratio
Artificial fill	Slightly dense	1.85	15	11	0.005	0.35
Clay	Hard plastic	1.90	35	17	0.010	0.35
Basalt	Full-weathered	1.85	100	22–23	0.01–0.04	0.30
Basalt	Strong-weathered	2.20	< 400	26–31	0.06–0.20	0.30
Basalt	Medium-weathered	2.50	400–800	31–35	0.20–0.40	0.28

### Construction method

For the shallow-buried tunnel of Class IV–VI surrounding rock under the existing highway project, when the tunnel is constructed by CRD method or CD method, the surrounding rock deformation control effect is good. But in actual construction, according to the principle of saving investment, speeding up the construction progress, and making the construction process as simple as possible, under the condition of strengthening the face and surrounding rock in advance (such measures as pipe-shed reinforcement in advance), the bench method or bench excavation method shall be selected as far as possible for the construction of shallow-buried mountain tunnel.

**Figure 3:**
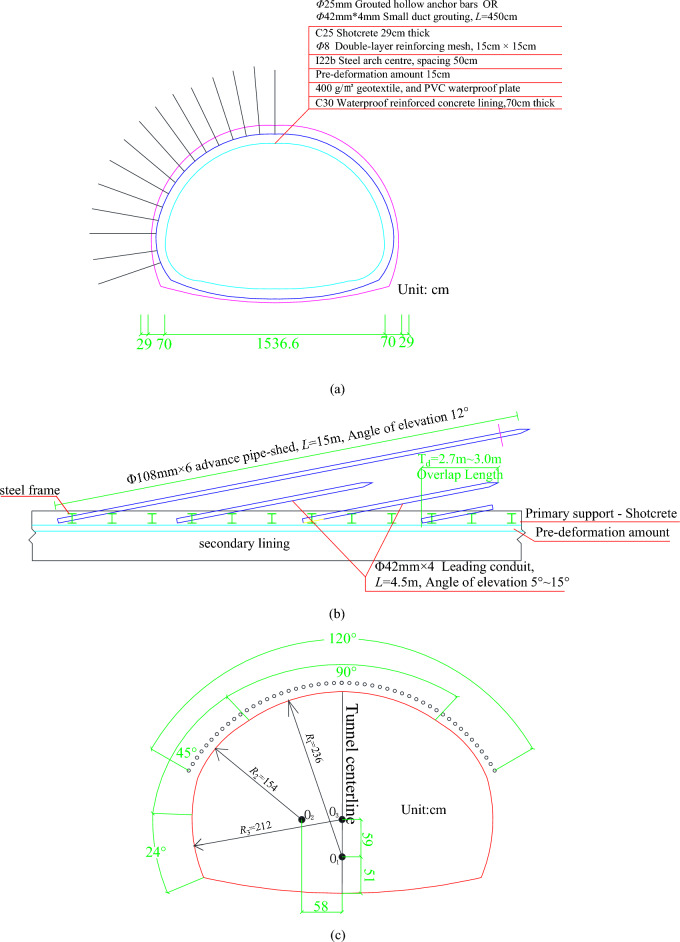
Tunnel support: (**a**) design drawing of support structure for tunnel crossing section; (**b**) vertical section diagram of advanced support measures; (**c**) layout plan for pipe-shed drilling.

The tunnel construction was carried out by mechanical excavation with the three-step method used in excavation. The tunnel excavation is 0.6–1.2 m per cycle, with the excavation sequence from right to left. As shown in Fig. 3, the advanced support plus primary support mode was adopted, in which the advanced support was pipe-shed pre-support, and the advanced pipe-shed is 15 m long Φ 76 mm × 4 hot-rolled seamless steel tubes with a circular spacing of 30 cm. The leading conduits are installed in about 120° of the lining arch, and the small conduit is made of Φ 42 mm × 4 hot-rolled seamless steel tubes with 5.0 m long and 40 cm circumferential spacing. The primary support consists of shotcrete (C25, thickness 29 cm), radial anchors (length 4.5 m, spacing 1.0 m * 0.6 m), reinforcing mesh (8@150 mm * 150 mm), and I-beam steel arch centre (spacing 50 cm).

## Three-dimensional stereoscopic monitoring system of existing highways under soft rock tunneling

### Monitoring program

**Figure 4:**
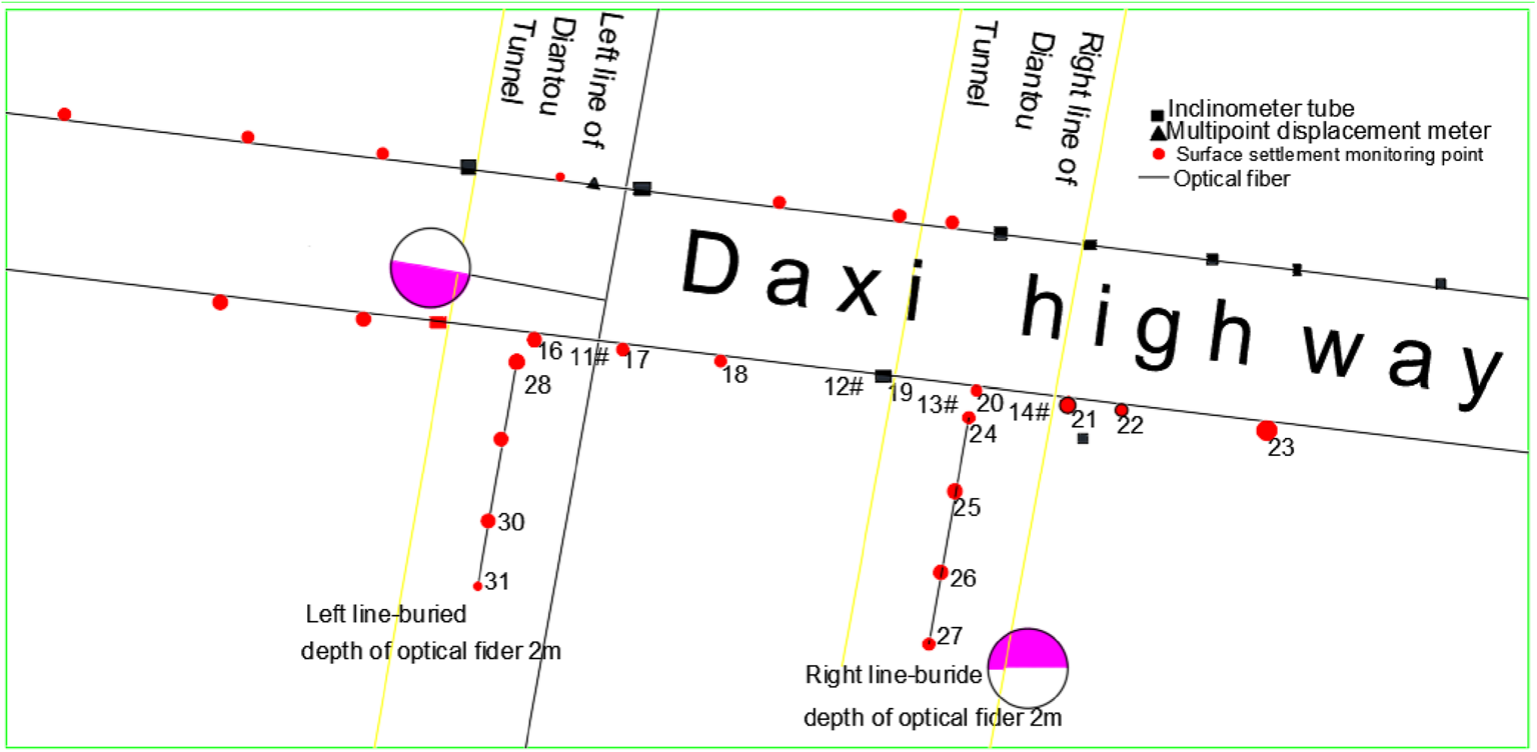
Distribution of monitoring sites.

By analyzing the surrounding environment of the tunnel, the burial conditions, and the actual traffic condition of the highway, the general arrangement of monitoring for the intersection section of the tunnel and the highway was determined, as shown in Fig. 4. Surface settlement monitoring points, tilt tubes and multi-point displacement gauges were placed along the roadside in the right line YK7 + 045 and YK7 + 028 sections, and in the left line ZK7 + 010 and ZK6 + 998 sections. Fiber optic sensors and surface settlement monitoring points were placed along the axis of the right vault of the tunnel in the right line YK7 + 025 to YK7 + 001 and in the left line ZK6 + 995 to ZK6 + 971 range. Internal structural stress monitoring sections were placed in the right tunnel YK7 + 038.9 and YK7 + 034.2, respectively.

As shown in Fig. 4, there are 31 surface settlement monitoring points and two reference points. In order to monitor the vertical deformation of the geotechnical layer of the cross-section of the highway, a high-resolution distributed optical fiber with a length of 24 m is arranged below the existing roadbed at a depth of about 2.0 m. Deep deformation monitoring mainly includes two parts, deep displacement and deep inclination measurement. Deep displacement was realized by setting the borehole with a multi-point displacement meter, and deep inclination measurement was realized by using the borehole with an inclination measurement tube. The plane position of the multi-point displacement meter and inclinometer is shown in Fig. 4. There are three multi-point displacement monitoring holes of 2#, 3#, and 10# in the left line, and three multi-point displacement monitoring holes of 6#, 7#, and 13# in the right line, totaling six multi-point displacement measuring holes. In the left line, there are four skew holes of 1#, 4#, 9#, and 11#, and 4 skew holes of 5#, 8#, 12#, and 14# in the right line, totaling eight skew holes. The bottom of the multi-point displacement meter hole is 0.5 m away from the top of the tunnel hole, and the bottom of the inclined hole is 1.0 m away from the base.

**Figure 5:**
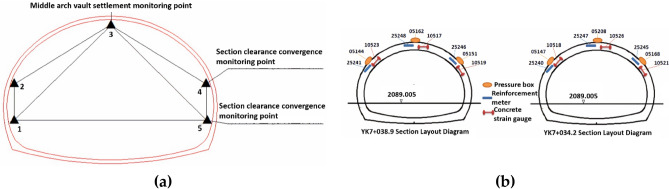
Monitoring section schematic: (**a**) monitoring points for arch crown settlement and convergence; (**b**) internal force monitoring points of surrounding rock and lining.

There are four monitoring sections^40,41^ for arch crown settlement and convergence in ZK6 + 998, ZK7 + 010, YK7 + 028, and YK7 + 045, with 5 points arranged in each section shown in Fig. 5a, including one arch crown settlement monitoring point and two displacement convergence monitoring points on each side. Four monitoring sections for the internal force of the surrounding rock and lining are arranged in the tunnel's cross-section, two sections for the left and right tunnel, YK7 + 038.9 and YK7 + 034.2, respectively, as shown in Fig. 5b.

### Monitoring frequency and warning

Considering that the site construction needs to pay particular attention to the road surface's settlement, the pavement settlement monitoring frequency is formulated according to the development speed of displacement and the distance from the excavation face. Moreover, the general principle is that the closer to the excavation face, the greater the displacement speed and the higher the monitoring frequency. As shown in Table 2, the monitoring frequency, the control criteria of steel support stress, surrounding rock pressure, concrete strain, horizontal deformation of surrounding rock, deep displacement of surrounding rock, and fiber optic monitoring, were adopted by the site implementation.

**Table 2:** Monitoring frequency and control standards for each monitoring project.

Monitoring item	Displacement speed	Distance from the working surface	Monitoring frequency	Total control amount (mm)	Controlling rate (mm/day)
Settlement of highway pavement	> 4.0 mm/day	(0.0–1.0)D	3 time/2 day	40	2.5
	1.0–3.0 mm/day	(1.0–2.0)D	1 time/day		
	0.5–1.0 mm/day	(2.0–5.0)D	1 time/2 day		
	< 0.5 mm/day	> 5.0D	1 time/7 day		
The stress of steel support			1 time/2 day		
Surrounding rock pressure					
Concrete strain					
Horizontal deformation of surrounding rock				25	0.2
Deep displacement of the surrounding rock				25	0.2
Fiber optic monitoring				40	2.5

## Analysis of monitoring results

### Arch crown settlement monitoring results and analysis

**Figure 6:**
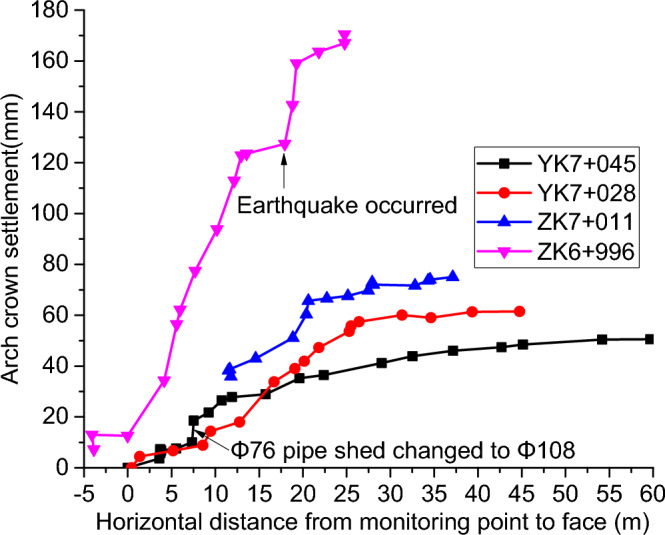
Settlement of arch crown.

Figure 6 shows the relationship between settlement and excavation at the arch vaults of sections YK7 + 045, YK7 + 028, ZK7 + 011, and ZK6 + 996. For the monitoring section YK7 + 045, the surface settlement only changed significantly after the excavation face passed through this section. Before that, YK7 + 050 on the right side of the tunnel, Φ 76 pipe-shed shall be reinforced. When the horizontal distance between the tunneling face and the monitoring point is about 7 m, the settlement of the arch crown will accelerate, and the change rate of deformation will obviously exceed the allowable value. The Φ 76 pipe-shed was changed to the Φ 108 pipe-shed, and then the road crossing section and the right side of the tunnel were reinforced with the Φ 108 pipe-shed to some extent. According to the monitoring results, it can be seen that the construction plan can effectively reduce the actual settlement change rate of the road crossing section YK7 + 045. During the stagnation period of 19.3 m from the excavation face at section ZK6 + 996, there was a large development of settlement at the top of the arch. This was caused by three consecutive earthquakes in Yangbi County, 29 days after the excavation of section ZK6 + 996, and the rainfall led to the softening of the soil^19^. On the one hand, earthquake action can cause disturbance to the undisturbed soil, leading to a decrease in soil strength^42–44^. On the other hand, the presence of seismic inertial forces can greatly weaken the stability of surrounding soil^45^. The pore water pressure and soil pressure increase with the duration of rainfall, and the greater the rainfall intensity, the greater the pore water pressure and soil pressure, which is more unfavorable for the stability of the surrounding soil^46^. Rainfall infiltration can reduce the effective cohesion and effective friction angle of the soil, which is not conducive to the stability of the surrounding soil^47^. After an earthquake, rainfall occurs, and the combined effect of the two greatly reduces the stability of the surrounding soil^48–50^. Also, the section was closer to the excavation face and more affected, thus causing the development of surface settlement at the top of the arch of section ZK6 + 996.

According to Fig. 6, the final surface settlement values at the arch crown of the above four sections were 51.0 mm, 61.0 mm, 76.0 mm, and 170.2 mm, respectively, which were mainly due to the shallower burial depth and worse geological conditions in the left tunnel than the right tunnel in the cross-section. When the tunneling face was excavated to the monitoring section, the cumulative deformation of surface settlement measurement points at the top of the arch reached 0–10% of the final deformation on average, after which the cumulative surface deformation tended to grow faster. The deformation change rate of the tunnel was significantly reduced after the completion of the inverted arch support structure. At the same time, although the settlement of each arch is still unavoidable, the accumulated deformation is maintained between 74 and 89% of the final deformation. The measured point subsidence reaches 90% of the final subsidence when the excavation face passes through about twice the diameter of the monitored surface (about 34 m), after which it maintains a slow growth and stabilizes. By analyzing the information contained in the diagram, it can be seen that the spatial constraint effect of the excavation face is obvious, covering up to about 25 m behind it, a distance of about 150% of the tunnel width.

### Surface settlement monitoring results and analysis

#### Lateral distribution of surface settlement

**Figure 7:**
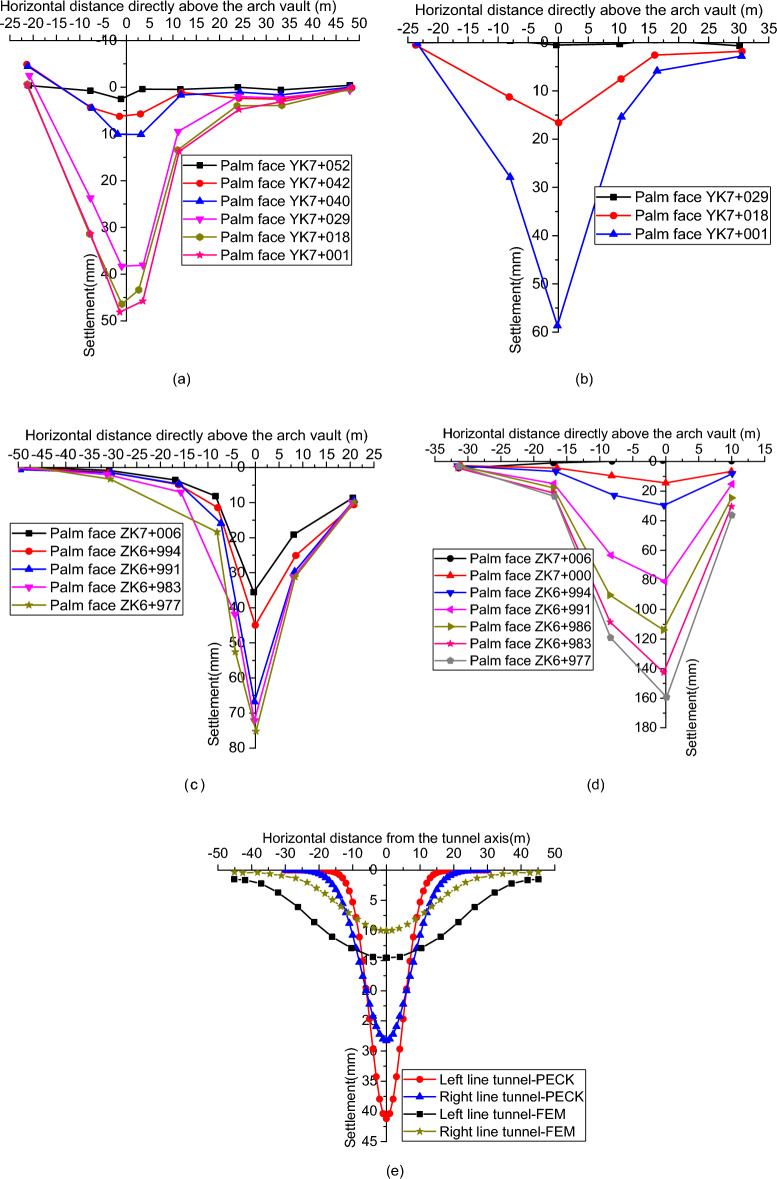
Transverse distribution of surface settlement: (**a**) YK7 + 045 horizontal position; (**b**) YK7 + 028 horizontal position; (**c**) ZK7 + 011 horizontal position; (**d**) ZK6 + 996 horizontal position; (**e**) Peck formula calculation results and FEM calculation results.

Figure 7a–d show the surface settlement change curves of sections YK7 + 045, YK7 + 028, ZK7 + 011 and ZK6 + 996, respectively. By analyzing the surface settlement trends shown in Fig. 7a–d, it can be seen that the surface settlement induced by the excavation tunnel is roughly funnel-shaped. Also, the settlement of its corresponding surface points from the top of the vault to the two sides gradually decreases, and the surface settlement on the outside of the two tunnels is smaller than that between them. By studying the range of surface settlement, it can be seen that if the central axis of the tunnel is taken as the reference, the most obvious area where the settlement deformation occurs on the left and right side of the surface above the tunnel is within the range of twice the diameter of the tunnel.

Peck's formula^51^ is the most commonly used formula for estimating surface deformation caused by tunnel excavation.

1 $$S(x) = S_{\max } \exp \left[ { - x^{2} /\left( {2i^{2} } \right)} \right]$$

2 $$S_{\max } = \frac{{V_{{{\text{loss}}}} }}{{i\sqrt {2\pi } }} = \frac{{\pi R^{2} \eta }}{{i\sqrt {2\pi } }}$$

where $x$ is the lateral horizontal distance from the tunnel axis; $S(x)$ is the amount of ground settlement; $i$ is the width coefficient of the ground settlement trough, with values of 4.938 m for the left line tunnel and 7.199 m for the right line tunnel; $V_{{{\text{loss}}}}$ is soil loss per unit length; $R$ is the tunnel excavation radius, and the value for this project is 7.356 m; $\eta$ is the soil loss rate, and the value for this project is 0.3%. For this project, the final ground surface settlement curve calculated according to the Peck formula is shown in Fig. 7e. The surface settlement calculated by the finite element method is also shown in Fig. 7e. Comparing the surface settlement curves in Fig. 7a–e, it can be clearly seen that both the measured results and the Peck formula calculation results show that the lateral ground surface settlement caused by tunnel excavation is in a groove shape.

The comprehensive analysis of the surface settlement deformation of YK7 + 045, YK7 + 028, ZK7 + 011, and ZK6 + 996 cross-sections shows that the distance between the two measurement points and the size of the settlement difference is one of the important reasons affecting whether cracks will appear between the two measurement points. The definition of transverse settlement deformation difference rate is the settlement difference between two measurement points/distance between two measurement points. Obviously, the number with a deformation difference rate greater than zero. The larger the transverse settlement deformation difference rate is, the more obvious the surface depression phenomenon will be, and the greater the possibility of tension cracks in a certain range on both sides of the tunnel axis. In addition, it is necessary to calculate the deformation difference rate in practice, which is because the traditional deformation control index does not entirely present the uniformity of settlement between two measurement points. In contrast, the uneven settlement will likely cause serious pulling crack damage to the road surface and other buildings. The deformation difference rate calculation is based on the traditional deformation control index. For example, the surface settlement needs to be monitored first, and then further steps such as screening and calculation are taken. Combined with the surface settlement data monitored in the field, the results of the transverse deformation difference rate of the four sections are easily obtained, as shown in Table 3. It can be seen that when the difference rate of transverse deformation of the ground surface is lower than 10 mm/m, the ground surface does not produce cracks, and when it reaches 11 mm/m, the ground surface produces cracks. Therefore, it is suggested that the control index of the difference rate of the ground surface transverse deformation can be set as 10 mm/m.

**Table 3:** Difference rate of ground surface transverse deformation.

Position	Difference rate of surface deformation (mm/m)	Surface cracks condition
YK7 + 045 left	2.87	No
YK7 + 045 right	4.08	No
YK7 + 028 left	3.57	No
YK7 + 028 right	4.09	No
ZK7 + 011 left	7.10	No
ZK7 + 011 right	5.30	No
ZK6 + 996 left	5.63	No
ZK6 + 996 right	11.27	Pulling crack at a roadside ditch

#### Longitudinal distribution of surface settlement

**Figure 8:**
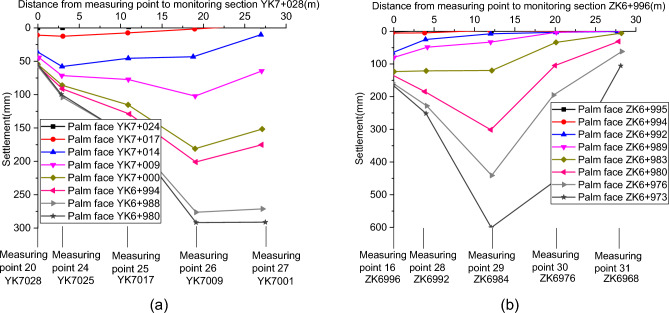
Longitudinal distribution of surface settlement: (**a**) right side of the tunnel; (**b**) left side of the tunnel.

Figure 8a shows the ground settlement curve of the longitudinal section from YK7 + 028 to YK7 + 001. When the excavation face passes point No. 24, the surface settlement of the right tunnel is generally large, with the main sinking points at monitoring points 26 and 27. The settlement of No. 24, No. 25, and No. 26 surface monitoring points increases rapidly after crossing YK7 + 014 on the excavation face of the tunnel. The differential settlement directly leads to cracks at the retaining wall of the corn field and the junction of the main hole cover of the Daxi Class II highway and the asphalt pavement. When the excavation face is located at YK6 + 980, the final cumulative settlement of monitoring points 20, 24, 25, 26, and 27 is 63.7 mm, 104.7 mm, 168.4 mm, 293.0 mm, and 295.5 mm, respectively.

Figure 8b shows the ground settlement curve for the longitudinal section from ZK6 + 996 to ZK6 + 968. Similarly, it can be seen that the settlement at monitoring points 16 and 28 stabilizes as the tunnel progresses. The settlement difference between monitoring points 16 and 28 is 30.3 mm when the excavation face is excavated to ZK6 + 980, and the settlement change rate and settlement difference between monitoring points 28, 29, and 30 near the steel plant is larger as the excavation face advances further, with obvious cracks in the road surface. The final settlement at monitoring points 28, 29, 30, and 31 was 256.2 mm, 603.7 mm, 453.2 mm, and 104.6 mm. Such large settlement change rates and cumulative settlement values resulted in the cracking of the surface of the steel plant and the overall depression of the area.

According to Fig. 8b, it can be seen that the axial surface above the tunnel between sections ZK6 + 996 and ZK6 + 968 also shows uneven settlement of different degrees. Multiple transverse cracks can be seen on the ground around the No. 16 measuring point. According to the analysis of monitoring data, the maximum longitudinal deformation difference rate value between No. 16–28 measuring points is 11.70 mm/m, while the value between No. 28–29 measuring points is 43.44 mm/m, and the maximum surface settlement is 603.7 mm. This maximum surface settlement value obviously does not meet the specification requirements. Site construction conditions, burial depth, and local geological conditions largely cause this. The burial depth of the left tunnel is only 10 m, and its lithology is weaker than that of the right tunnel.

Furthermore, the thickness of the artificial backfill around the ZK6 + 996 section is about twice the thickness of the rock layer. The former is about 7 m, while the latter is only about 3 m. The top of the ZK6 + 968 section is all artificial backfill. Considering the actual situation of the site construction, it can be found that the constructor increased the amount of excavation face advancement at section ZK6 + 984 in order to advance the project progress as soon as possible. Furthermore, the excavation face was advanced too fast before the primary support strength reached the requirement, eventually leading to the surrounding rocks' severe deformation around section ZK6 + 984. After discovering this serious problem, the workers are suggested to adopt three-step of reserved core soil method for subsequent excavation, strengthen the site's support strength and close the excavation face in time.

In summary, when uneven settlement occurs along the axial and lateral directions of the tunnel and the difference between longitudinal and lateral deformation is large in actual engineering, there will be more obvious tension cracks in the corresponding area. In order to avoid cracks on the surface and on the highway to the greatest extent, the construction policy should strictly control the lateral deformation difference rate and longitudinal deformation difference rate within 10 mm/m for the shallow-buried tunnel with surrounding rock grade V. The deformation of the surrounding rock of large-section shallow-buried soft tunnel is largely affected by construction progress, buried depth, and lithology of surrounding rock. Once the deformation of the surrounding rock exceeds the specified value of engineering safety standards during construction, it is more suitable to adopt the CRD method or three-step reserved core soil rule.

After analyzing the distributed fiber optic monitoring results, it is found that the distributed fiber optic monitoring settlement results and the surface settlement monitoring results are basically the same in distribution pattern. The overall fiber optic displacement monitoring results are slightly smaller than the surface settlement monitoring results, which own to the settlement results of distributed fiber monitoring relative to the settlement at YK7 + 025 and ZK6 + 995.

### Results and analysis of deep displacement monitoring of the surrounding rock

**Figure 9:**
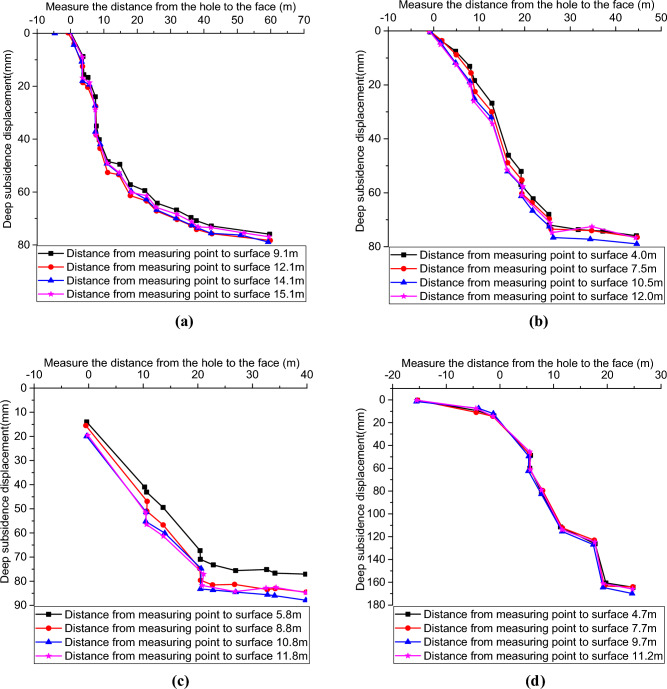
Deep displacement of surrounding rock: (**a**) 7 # hole; (**b**) 13 # hole; (**c**) 2 # hole; (**d**) 10 # hole.

Actually, the actual vertical displacement inside the surrounding rock is the sum of surface settlement and multi-point displacement meter readings. Accordingly, the vertical displacement curves of the surrounding rock of the 7# hole at YK7 + 045, 13# hole at YK7 + 028, 2# hole at ZK7 + 011 and 10# hole at ZK6 + 996 in the right line can be derived as shown in Fig. 9. It can be seen that with the tunnel boring, the internal sinking of the surrounding rock occurs to different degrees and eventually stabilizes. More precisely, the sinkage difference between 9.1 m and 12.1 m of hole 7# from the surface is 3.26 mm, and the maximum deep sinkage is 76.29 mm, which occurs at 12.1 m burial depth. While the sinkage difference between 4.0 and 10.5 m of for hole 13# from the surface is 5.05 mm, and the maximum deep sinkage is 79.61 mm, which occurs at 10.5 m burial depth. The sinkage difference of hole #2 between 5.8 and 11.8 m from the surface is 7 mm, and the sinkage of 11.8 m from the surface is 84 mm. And the sinkage of surrounding rocks of hole #10 at different burial depths is basically the same, and the final stabilized sinkage is 169.3 mm. Overall, the order of deep displacement in the four sections is YK7 + 045 < YK7 + 028 < ZK7 + 011 < ZK6 + 996. The sinking pattern of the deep surrounding rock in each monitoring hole is not the same, which is related to the geological conditions of the site. Since the 7 # hole at section YK7 + 045 and 2 # hole at section ZK7 + 011 are close to the inner side of the mountain, the surrounding rock conditions are generally better than the other two sections, and the buried depth of the right tunnel is about 14 m, while the buried depth of the left tunnel is about 10 m. The tunnel undercrossing construction process has a greater impact on the existing highway above the shallow buried depth.

### Monitoring results and analysis of surrounding rock inclinometer

**Figure 10:**
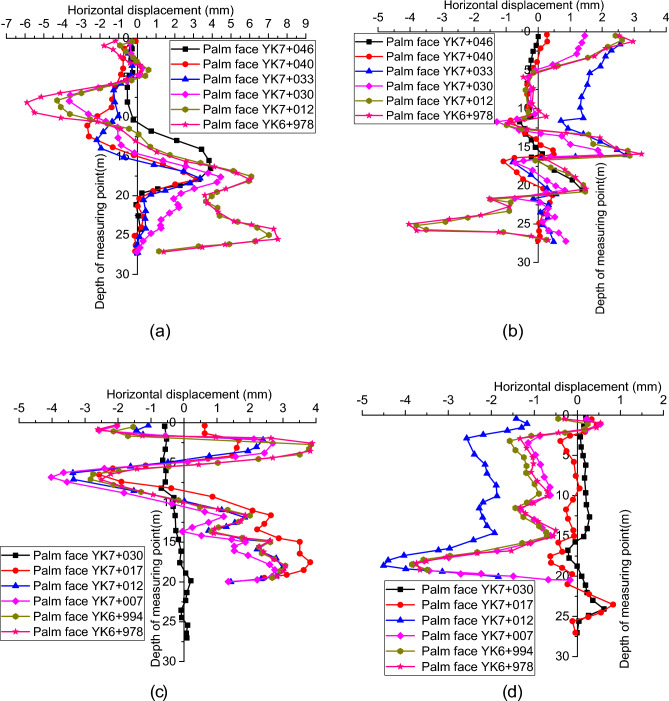
Inclination measurement results of surrounding rock: (**a**) 5 # hole at YK7 + 045 section (left side of the tunnel); (**b**) 8 # hole at YK7 + 045 section (right side of the tunnel); (**c**) 12 # hole at YK7 + 028 section (left side of the tunnel); (**d**) 14 # hole at YK7 + 028 section (right side of the tunnel).

Four inclined holes in the left line were blocked at different depths and could not be tested owing to construction. And 05# and 8# holes at the YK7 + 045 section and 12# and 14# holes at the YK7 + 028 section all measured the data smoothly, as shown in Fig. 10.

The results of inclination monitoring showed that the surrounding rock at the YK7 + 045 section was deformed to different degrees. When the excavation face of the right tunnel is excavated to the right section YK6 + 977.6, that is, when it exceeds 67.4 m of the intersection (the right section YK7 + 045) of Daxi Road and Diantou Tunnel, from the deformation results of the 5 # hole on the left side and the 8 # hole on the right side of the tunnel, obvious lateral horizontal deformation occurs on both sides of the tunnel with a buried depth of 15–20 m and 25 m. The direction points to the tunnel excavation side. When the tunnel excavation's upper, middle, and lower benches are free, obvious lateral horizontal deformation occurs in the surrounding rock of the side wall.

Similarly, for the inclinometer monitoring section YK7 + 028, when the excavation face of the right tunnel is excavated to the right section YK6 + 977.6, which is more than 50.4 m from the intersection (the right section YK7 + 028) of Daxi Road and Diantou Tunnel, the main lateral horizontal deformation is at the depths of 5–10 m and 15–20 m, which corresponds to the free section of the first and second steps about 8 m above and below the tunnel crown side, that is, there is lateral horizontal deformation after the tunnel excavation free. As shown in Fig. 10d, the 14 # inclinometer hole is deformed, and the surrounding rock at the right side of the tunnel with a depth of 15–20 m has obvious lateral horizontal deformation.

### Monitoring results and analysis of tunnel arch crown settlement and convergence

**Figure 11:**
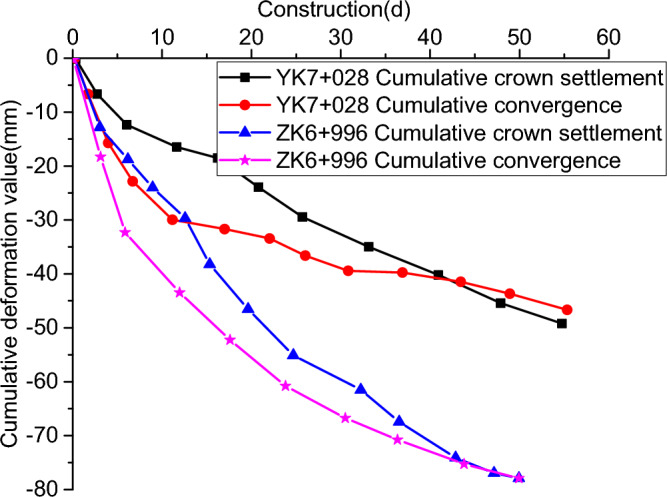
Tunnel arch crown settlement and convergence value curve.

Figure 11 shows the time curves of arch crown settlement and convergence at section YK7 + 028 and section ZK6 + 99. It can be seen that the arch crown settlement and convergence increased rapidly in the first 10 d, and the sinking rate decreased significantly after 40 d after bolting and shotcreting, while the deformation was slow and in a stable state after about 50 d. Where the final arch crown settlement and convergence values of section YK7 + 028 are 49 mm and 46 mm, respectively, and the final arch crown settlement and convergence of section ZK6 + 996 are 77 mm and 78 mm, respectively, and the arch crown settlement and convergence values of the left section are obviously larger than those of the right section.

### Monitoring results and analysis of tunnel arch crown settlement and convergence

**Figure 12:**
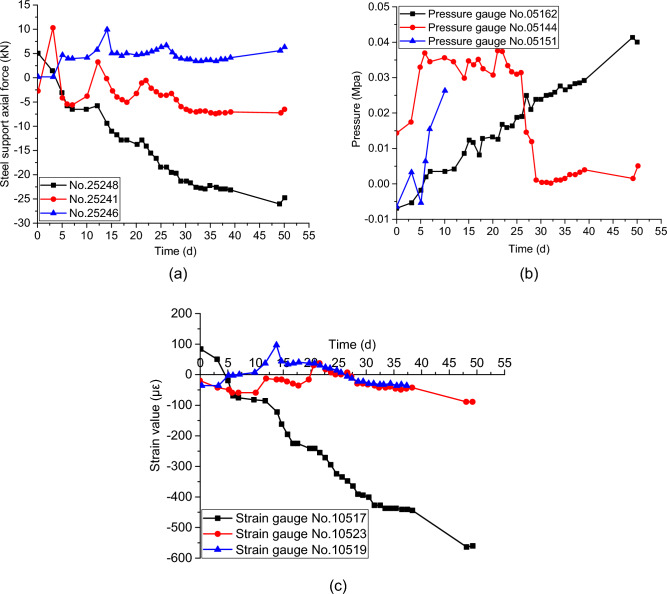
Monitoring results for the primary support structure of the surrounding rock at section YK7 + 038.9: (**a**) steel support axial force; (**b**) Surrounding rock pressure; (**c**) concrete strain.

Two sections were set up for stress monitoring of surrounding rock and support structure, namely YK7 + 038.9 and YK7 + 034.2. The following is a specific analysis of the section K7 + 038.9. From Fig. 12a, it can be seen that the internal force values of the three steel reinforcement meters gradually increase, indicating that as the excavation face advances, the steel support is gradually bearing the pressure from the upper surrounding rock. The maximum peak internal force of the steel support in the section K7 + 038.9 reaches -25.98KN, and corresponding to the steel reinforcement meter No. 25248. From Fig. 12b, it can be seen that the maximum peak pressure of the surrounding rock reaches 0.0416 Mpa, located at the arch crown, and its pressure shows a linear increasing trend, indicating that the arch crown is greatly affected by the surrounding rock pressure. The other two pressure gauges show that the pressure first increases and then decreases, indicating that the arch shoulder is slightly less affected by the compression of the surrounding rock compared to the arch crown. From Fig. 12c, it can be seen that the overall trend of the three curves is consistent, indicating that the arch is under overall compression, and the compressive stress at the arch crown is the highest. According to the concrete strain gauge No. 10517 on the arch crown, the maximum peak strain at the corresponding position has reached − 558.5 με, obviously greater than 300 με. When the elastic modulus of the primary support concrete was set to 2 Gpa, the strain at the corresponding position was converted to a stress of 1.117 Mpa, indicating that the secondary lining may be under certain pressure. As the excavation of the upper, middle, and lower benches passes through this monitoring section, there are significant changes in the monitoring values of the steel reinforcement gauge, surrounding rock pressure gauge, and concrete strain gauge. Ultimately, the development trend of each measuring point is basically stable, indicating that the combination of surrounding rock and support structure is relatively sufficient. In terms of construction technology, in order to better control the surface settlement of highways, it is necessary to adopt Φ 108 pipe-shed replacement Φ 76 pipe-shed for tunnel reinforcement and encrypt arch supported steel structure with tunnel cross-section, which better consolidated the reinforcement effect.

## Relationship between surrounding rock deformation and advanced support measures, deformation index suggestions

### Relationship between the deformation of the surrounding rock and the advanced supporting measures

**Figure 13:**
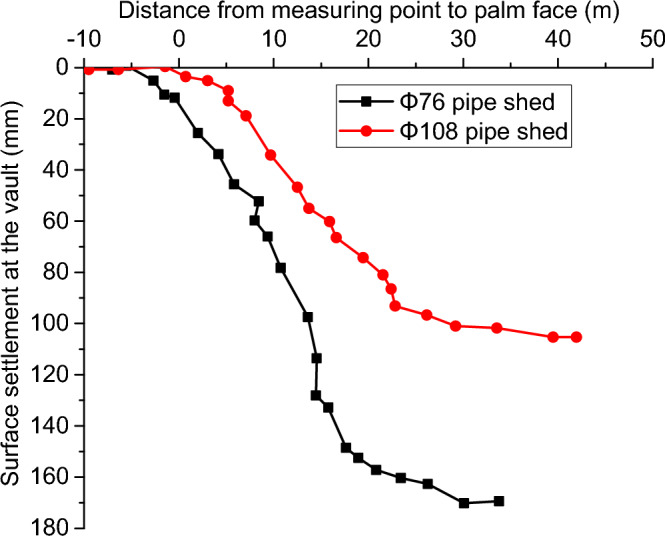
Relationship between the surface settlement of the arch vault and the distance of the measurement point from the excavation surface.

In Fig. 4, the 24 # surface monitoring point and 25 # surface monitoring point at the crown of the right line have relatively close geological conditions, but the 24 # and 25 # sections are, respectively, Φ 76 and Φ 108, two different pipe-shed supports. Figure 13 shows the relationship curve between the distance from the measuring point to the excavation face and the surface settlement of the arch crown under the two different pipe-shed pre-supports. It can be seen that during the continuous excavation of the right excavation face of the tunnel and the gradual approach to the surface measurement point, the surface deformation of the arch vault under Φ 76 pipe-shed pre-support is more obvious than that under Φ 108 pipe-shed pre-support, with the surface settlement of the former is 12.5 mm, accounting for 7.3% of the final settlement. While the surface settlement of the latter is only 2.9 mm, accounting for 2.7% of the final settlement. Compared with the Φ 76 pipe-shed, the Φ 108 pipe-shed controls the surface settlement change rate and amount of settlement and reduces the maximum surface settlement of the arch vault by 38%, thus maintaining the stability of the surrounding rock. According to the actual monitoring results, the Φ 108 pipe-shed solution is more effective than the Φ 76 pipe-shed solution in the special working condition of a shallow buried soft tunnel with a large section.

**Figure 14:**
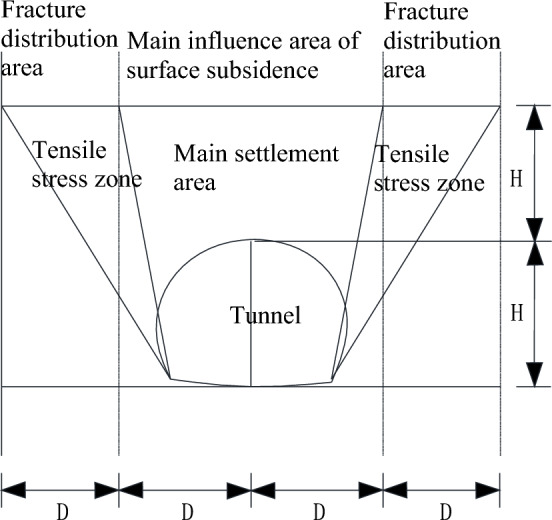
Surface fracture distribution prediction map.

Based on the previous analysis of transverse deformation differences, longitudinal deformation differences, and the study of the above main influencing factors, the prediction diagram of surface fissure distribution, as shown in Fig. 14, is made for the influence of shallow tunnel construction on surface settlement under the condition of overlying surrounding rock, which is mainly composed of a loose layer. Moreover, the tunnel buried depth equals the chamber height, which can provide a reference for monitoring and early warning of similar projects.

### Control index of rock deformation in a tunnel

Take the four transverse sections YK7 + 045, YK7 + 028, ZK7 + 011, and ZK6 + 998 on both sides of the highway crossing section and the two longitudinal sections YK7 + 025 ~ YK7 + 001, ZK6 + 995 ~ ZK6 + 971 on the tunnel and highway crossing section for comparative analysis in combination with the site surface monitoring data and construction safety conditions. The summary of surface settlement monitoring data is shown in Table 4.

**Table 4:** Summary of surface settlement data at major monitoring sites.

Position	Settlement (mm)	Settlement change rate (mm/day)	Deformation difference rate (mm/m)	Surface cracks	Site conditions
Transverse YK7 + 045	51.00	3.22	4.08	Non	Depth 14.00 m
Transverse YK7 + 028	61.00	9.00	4.09	Non	Depth 14.00 m
Transverse ZK7 + 011	76.00	11.30	7.10	Non	Depth 10.00 m
Transverse ZK6 + 988	170.20	26.80	11.27	Multiple	Depth 10.00 m, poor surrounding rock
Portrait YK7 + 025 ~ 7 + 011	295.50	27.00	15.58	Multiple	Poor surrounding rock, weak advanced support
Portrait ZK6 + 995 ~ 6 + 971	603.70	29.30	43.44	Multiple	Poor surrounding rock, weak advanced support, fast tunneling

By comparing the monitoring and control indexes of similar tunnels and combining with the results of monitoring deformation of existing highways under tunnels, the following suggestions are given for the safety control indexes of tunnel deformation: For the shallow tunnel undercrossing the existing important buildings and structures, when the accumulated deformation of the deep surrounding rock of the section is less than 50 mm and the change rate is less than 2 mm/day, the ground surface basically does not produce cracks, which is basically consistent with the specification control index; when the cumulative deformation is 50–80 mm, and the change rate is 3–6 mm/day, the surface may produce cracks; When the cumulative deformation exceeds 100 mm and the change rate is greater than 8 mm/day, the corresponding surface is likely to produce cracks, as shown in Table 5.

**Table 5:** Suggested control indexes for surface settlement.

Monitoring items	Surrounding rock grade	Allowable settlement (mm)	Allowable settlement change rate (mm/day)	Allowable settlement difference rate (mm/m)	Corresponding security status
Surface subsidence	V	< 50.00	< 3.00	< 5.00	No crack
		50.00–100.00	3.00–6.00	5.00–10.00	Crack may occur
		> 100.00	> 6.00	> 10.00	Cracks are likely to occur

## Discussion

In the pipe-shed grouting method, the pipe-shed reinforces the surrounding rock through grouting, the loose surrounding rock above the arch is reinforced and compacted to form a solid structure, increasing overall stability and forming a plate and shell effect to a certain extent^36^. Along the transverse direction of the tunnel, the pipe-shed and surrounding rock jointly form a certain thickness of circumferential bearing structure, which plays a role in advanced support and jointly bears the load from the surrounding rock due to the tunnel excavation. Along the axial direction of the tunnel, the advanced pipe-shed and the primary support structure form a bearing system for bearing and play the role of beam support, which can effectively control the relaxation deformation and stress release of the surrounding rock above the tunnel, thereby improve the stability of the surrounding rock from the excavation face and avoid instability and collapse of the excavation face^37^. The greater the stiffness of the pipe-shed support, the greater the overlying load it bears. By reducing the relaxation deformation and stress release of the upper surrounding rock, the deformation of the surrounding rock can be effectively controlled^31,34^. The deeper the burial depth, the easier it is for the three-dimensional arch effect to form near the excavation surface. The formation of pressure arches helps to maintain the stability of tunnel excavation face and can reduce surface settlement to a certain extent^39^.

It should be noted that the relationship between surface settlement and cracks shown in Tables 4 and 5 is derived for V-grade surrounding rock. The strength of surrounding rock varies in different grades, and the relationship between surface settlement and cracks is theoretically different. It is recommended to use more engineering examples in the future and learn from the research method in this paper to statistically analyze the relationship between cracks and settlement under different grades of surrounding rock, in order to better guide engineering practice.

## Conclusion

Through three-dimensional stereoscopic monitoring of a specific engineering example of a shallow-buried soft rock tunnel crossing a highway, the evolution laws of the tunnel structure's stress, deformation, deep displacement of surrounding rock, and impact range over time and space during the entire process of tunnel construction were obtained, and the impact mechanism of shallow-buried soft rock tunnel excavation on the upper existing highway, as well as the mechanism of pipe-shed reinforcement, were clarified. The main conclusions are as follows:

1. Construction methods, topographic features, and actual geological conditions are the main factors that affect the deformation of surrounding rock. Large diameter pipe-shed advanced pre-support can effectively reduce the impact of shallow soft rock tunnel excavation on the upper existing highway.
2. From the cross-sectional analysis of the tunnel, when the tunnel is constructed under the highway, the approximate range of the main impact area of surrounding rock deformation is about one time the diameter distance, and the approximate range of the overall impact area is about two times the diameter distance; From the longitudinal section analysis of the tunnel, the surrounding rock deformation begins to be relatively stable when the distance between the tunneling face and the monitoring section is about twice the tunnel diameter.
3. For shallow tunnels in Class V surrounding rock, the two settlement control indicators, the change rate of settlement deformation and total deformation amount, cannot directly reflect the situation of surface cracks. The newly proposed deformation difference rate can effectively reflect the situation of surface cracks. It is suggested that multiple indicators such as settlement deformation change rate, total deformation, lateral deformation difference rate, and longitudinal deformation difference rate can be comprehensively used for settlement deformation control in practical projects.
4. The safety control index of V-class shallow buried tunnel surrounding rock deformation: when the accumulated deformation of deep surrounding rock is less than 50 mm, the change rate is less than 3 mm/day, and the deformation difference is less than 5 mm/m, there is no crack on the surface. When the accumulated deformation is at 50–100 mm, the change rate is 3–6 mm/day, and the difference rate reaches 5–10 mm/m, cracks may occur at the surface. When the cumulative deformation exceeds 100 mm, the change rate is greater than 6 mm/day, and the difference rate is greater than 10 mm/m, the corresponding surface is likely to produce cracks.

This paper uses a combination of theoretical analysis and on-site monitoring methods to study the impact mechanism of shallow soft rock tunnel excavation on the upper existing highway and the reinforcement mechanism of pipe-shed. However, different projects have different characteristics. How to optimize the design of pipe-shed for similar projects while ensuring reasonable ground settlement is a research direction with great engineering application prospects.

## Data Availability

Some or all data that support the findings of this study are available from the corresponding author upon reasonable request.
